# 1485. Landscape of peripheral blood mononuclear cell single-cell transcriptomes in HIV-infected patients with cryptococcal meningitis

**DOI:** 10.1093/ofid/ofad500.1321

**Published:** 2023-11-27

**Authors:** Yaokai Chen, Yao Li, Ting Zhao

**Affiliations:** Chongqing Public Health Medical Center, chongqing, Chongqing, China; Chongqing Public Health Medical Center, chongqing, Chongqing, China; Chongqing Public Health Medical Center, chongqing, Chongqing, China

## Abstract

**Background:**

Cryptococcal meningitis (CM) is a lethal opportunistic infection in patients with human immunodeficiency virus (HIV); however, the immunopathogenic mechanisms underlying HIV-associated cryptococcal meningitis (HIV-CM) remain largely unknown.

**Methods:**

Here, using the single-cell nucleus sequencing approach, we compared peripheral blood mononuclear cell (PBMC) transcriptomic alterations among ART-naïve HIV-CM patients (N=8), ART-naïve HIV-infected individuals (N=8), and healthy controls (HC, N=8). Additionally, alteration of the single-cell transcriptomes of HIV-CM patients before and after four-week antifungal treatment was also analyzed, to estimate the influence of treatment.

**Results:**

In total, we obtained 318,718 PBMCs and identified 20 cell subclusters based on gene expression. In our PBMC sample, we observed the significantly decreased percentage of CD4+ T-cells and NK cells, as well as an increased percentage of CD14 monocytes in HIV-CM patients, relative to HC. Only the percentage of CD4+ T-cells was significantly altered in HIV-CM patients, compared to HIV-infected individuals. Moreover, after four-week of antifungal treatment, the percentage of these three cell types were significantly altered. After treatment, the proportion of CD4+ T-cells in HIV-CM patients was observed to be lower compared to HC, but was comparable to HIV-infected individuals. The relative fractions of NK and CD14 monocytes were also reversed, to revert to normal levels. The functions of the HIV-CM-specific differentially expressed genes (DEGs) among the three cell types are related to cytoplasmic translation, signal recognition particle (SRP)-dependent co-translational protein targeting, viral processes, and others. After antifungal treatment, the rate of reversal of the DEGs ranged from 11.7% to 22.9%. The reversed genes are mainly associated with neutrophil degranulation and immune system processes.

**Conclusion:**

Together, the preceding findings suggest that the degree of change to the transcriptomes of PBMCs may be utilized as a potential marker for HIV-CM patients and for functional assessment of antifungal treatment. Furthermore, the disturbed molecular pathways may aid in understanding fundamental immunopathogenic pathways inherent to HIV-CM patients.

**Disclosures:**

**All Authors**: No reported disclosures

